# Neuronal Adenosine A2A Receptors Are Critical Mediators of Neurodegeneration Triggered by Convulsions

**DOI:** 10.1523/ENEURO.0385-18.2018

**Published:** 2018-12-26

**Authors:** Paula M. Canas, Lisiane O. Porciúncula, Ana Patrícia Simões, Elisabete Augusto, Henrique B. Silva, Nuno J. Machado, Nélio Gonçalves, Tiago M. Alfaro, Francisco Q. Gonçalves, Inês M. Araújo, Joana I. Real, Joana E. Coelho, Geanne M. Andrade, Ramiro D. Almeida, Jiang-Fan Chen, Attila Köfalvi, Paula Agostinho, Rodrigo A. Cunha

**Affiliations:** 1 CNC-Center for Neuroscience and Cell Biology, University of Coimbra, 3004-517 Coimbra, Portugal; 2 Department of Neurology, Boston University School of Medicine, Boston, MA 02118; 3 Faculty of Medicine, University of Coimbra, 3004-504 Coimbra, Portugal

**Keywords:** adenosine, convulsions, neuroprotection, synapse, synaptotoxicity, synatic plasticity

## Abstract

Neurodegeneration is a process transversal to neuropsychiatric diseases and the understanding of its mechanisms should allow devising strategies to prevent this irreversible step in brain diseases. Neurodegeneration caused by seizures is a critical step in the aggravation of temporal lobe epilepsy, but its mechanisms remain undetermined. Convulsions trigger an elevation of extracellular adenosine and upregulate adenosine A_2A_ receptors (A_2A_R), which have been associated with the control of neurodegenerative diseases. Using the rat and mouse kainate model of temporal lobe epilepsy, we now tested whether A_2A_R control convulsions-induced hippocampal neurodegeneration. The pharmacological or genetic blockade of A_2A_R did not affect kainate-induced convulsions but dampened the subsequent neurotoxicity. This neurotoxicity began with a rapid A_2A_R upregulation within glutamatergic synapses (within 2 h), through local translation of synaptic A_2A_R mRNA. This bolstered A_2A_R-mediated facilitation of glutamate release and of long-term potentiation (LTP) in CA1 synapses (4 h), triggered a subsequent synaptotoxicity, heralded by decreased synaptic plasticity and loss of synaptic markers coupled to calpain activation (12 h), that predated overt neuronal loss (24 h). All modifications were prevented by the deletion of A_2A_R selectively in forebrain neurons. This shows that synaptic A_2A_R critically control synaptic excitotoxicity, which underlies the development of convulsions-induced neurodegeneration.

## Significance Statement

Epilepsy is an evolving disease where neurodegeneration is associated with the aggravation of subsequent convulsions. We now unveil that the upregulation of adenosine A_2A_ receptors (A_2A_R) is paramount to link convulsions to neurodegeneration. This involves a translation of A_2A_R within synapses to bolster the activity of excitatory glutamatergic synapses and triggers an excitotoxicity first of synapses, that later evolves into neurodegeneration through increased calpain activity. Accordingly, the pharmacological or genetic blockade of A_2A_R arrested neurodegeneration, thus prompting A_2A_R as novel targets to alleviate neuronal damage associated with epilepsy.

## Introduction

Sclerotic temporal lobe epilepsy is the most common type of seizure disorder; it is an evolving disease phenotypically characterized by episodes of tonic-clonic convulsions that trigger adaptive changes and damage of brain tissue causing an aggravation of convulsions over time (Pitkänen and Sutula, 2002). Although this seizure-induced neurodegeneration is a key component of the pathophysiology of epilepsy, the underlying mechanisms are still unclear (Löscher and Brandt, 2010). One proposed participant in epileptogenesis is the adenosine modulation system (Dragunow, 1988; Dunwiddie, 1999; Boison, 2016): increased neuronal activity, and convulsions in particular, triggers a robust (During and Spencer, 1992) and sustained (Berman et al., 2000) elevation of the extracellular adenosine levels. Inhibitory adenosine A_1_ receptors (A_1_R) are considered a major anti-convulsive system, since the acute administration of A_1_R agonists decreases seizures whereas the acute administration of A_1_R antagonists worsens seizures and the consequent neuronal damage (Dragunow, 1988; Dunwiddie, 1999). However, inhibitory A_1_R appear to have a limited time window of effectiveness since there is a decreased density and a reduced efficiency of A_1_R in afflicted brain regions after the induction of seizures (Young and Dragunow, 1994; Ochiishi et al., 1999; Rebola et al., 2003; but see Gouder et al., 2003). This matches the role of A_1_R as “gate-keepers” of brain tissue viability, since their activation increases the threshold for brain damage; however, A_1_R undergo a reduction of their density and efficiency on chronic brain dysfunction (Cunha, 2005). Accordingly, A_1_R efficiently control the spreading of ictal events to “naïve” regions (Fedele et al., 2006; Zeraati et al., 2006) and A_1_R lose efficiency over time to control neurodegeneration (von Lubitz, 2001; Cunha, 2016).

By contrast, the density of adenosine A_2A_ receptors (A_2A_R) increases in limbic regions in different experimental models of epilepsy (Rebola et al., 2005a), as well as in sclerotic regions of patients with temporal lobe epilepsy (Barros-Barbosa et al., 2016). Cortical A_2A_R are mostly located in synapses (Rebola et al., 2005b) but also in glia (Orr et al., 2009, 2015; Rebola et al., 2011), and their activation increases glutamate release (Lopes et al., 2002; Marchi et al., 2002), enhances NMDA receptor function (Rebola et al., 2008), and bolsters neuroinflammation (Rebola et al., 2011) in the hippocampus. This provides a mechanistic basis for the robust neuroprotection afforded by A_2A_R antagonism in different noxious brain conditions (Chen et al., 2007; Cunha, 2016). Accordingly, the genetic deletion of A_2A_R slows down epileptogenesis (El Yacoubi et al., 2001, 2009), but the role of A_2A_R in the control of convulsions-induced neurodegeneration is still unclear (Lee et al., 2004; Rosim et al., 2011; Li et al., 2012).

This was now probed using kainate to trigger an acute convulsive period leading to subsequent neurodegeneration (Sperk et al., 1983; Coyle, 1987), modeling temporal lobe epilepsy (Ben-Ari, 1985). We report that the pharmacological or genetic blockade of A_2A_R did not affect kainate-induced convulsions but dampened the subsequent neurotoxicity, which begins with maladaptive alterations in synapses.

## Materials and Methods

### Animals

Male Wistar rats and C57Bl/6 mice (8–10 weeks) were from Charles River. C57BL6 global A_2A_R-knock-out (KO) mice and forebrain A_2A_R-KO mice were generated as previously described (Shen et al., 2008). Rodents were handled following European Union Directives (2010/63) on approval by the Ethical Committee of the Center for Neuroscience and Cell Biology (Orbea 78-2013).

### Kainate administration and evaluation of convulsions

In rats, kainate (Tocris) was injected intraperitoneally at a dose of 10 mg/kg, following our previous experience (Rebola et al., 2005a). The selective A_2A_R antagonist SCH58261 (Tocris) was used at an effective dose of 0.05 mg/kg (Lopes et al., 2004), administered intraperitoneally 30 min before kainate in most experiments, or 4 h after the extinction of convulsions in the last experimental protocol. A_2A_R-KO and wild-type littermates or forebrain A_2A_R-KO and floxed A_2A_R-KO littermates (designated as wild-type), all with a C57BL6 background, were injected subcutaneously with either saline or kainate (35 mg/kg). The animals were observed for 3 h to score convulsions (Rebola et al., 2005a) according to the original Racine scale for rats or the Racine scale adapted to mice and then maintained in groups of three to four per cage.

### Amygdala kindling

Amygdala kindling involved three groups: control, fully kindled and sham-operated rats. After one to two weeks of inserting an electrode in the amygdala, rats were stimulated twice a day (10 A.M. and 4 P.M.) with a 1-s train at 50 Hz with pulses of 1 ms and 500 µA, as previously described (Rebola et al., 2005a). After 24 d of stimulation, vehicle-treated rats were considered fully kindled (five consecutive sessions reaching stage 4–5). SCH58261 (0.05 mg/kg) was administered intraperitoneally twice a day, 30 min before each stimulation (or handling).

### Neuronal damage, astrogliosis, and microgliosis

The rodents’ brain was sectioned into 20-µm-thick coronal sections to analyze the general neuronal morphology using a cresyl violet staining of Nissl bodies (Kaster et al., 2015), cell damage using FluoroJade-C staining (Kaster et al., 2015), astrocytosis using GFAP immunoreactivity (Rebola et al., 2011; Kaster et al., 2015) with Cy3-conjugated anti-GFAP mouse antibodies (Sigma, 1:500), and microgliosis using either OX-42/CD11b immunohistochemistry (Rebola et al., 2011; Kaster et al., 2015) with mouse anti-CD11b antibodies (1:200; Serotec) or labeling with biotin-labeled tomato lectin (Dalmau et al., 2003; Vector Laboratories, 1:250). The integral of staining of FluoroJadeC (reporting degenerated neurons), CD11b (staining microglia), or GFAP (staining astrocytes) was counted in three regions of 50 × 50 μm in three sections per animal. These windows were selected free hand and placed in the stratum pyramidale (FluoroJadeC) or stratum radiatum (GFAP, CD11b) of the CA1 or CA3 regions, and a measure was taken in the anterior commissure as background.

To assess the localization of A_2A_R in microglia-like cells, brain sections from saline- or kainate-treated mice were collected 24 h after kainate injection and incubated with a combination of mouse anti-A_2A_R (1:500; Millipore Biotechnology) and rabbit anti-CD11b (1:100; Serotec) antibodies, followed by incubation with Alexa Fluor-labeled secondary antibodies (1:200; Invitrogen). Nuclei were stained with Hoechst 33342 (2 μg/ml) and sections were analyzed in a confocal microscope (LSM510; Zeiss).

### Exposure of synaptosomes to kainate

Hippocampal synaptosomes (purified synapses), prepared as previously described (Rebola et al., 2005b; Canas et al., 2014; Kaster et al., 2015), were incubated for 2 h at 37°C in the absence or presence of kainate (5 μM), without or together with cycloheximide (100 μM).

### Receptor binding assay

The density of A_2A_R was estimated by radioligand binding assays using a supramaximal concentration of ^3^H-SCH58261 (6 nM; offered by E. Ongini, Schering-Plow, Italy), as previously described (Rebola et al., 2005b; Kaster et al., 2015). Specific binding was determined by subtraction of non-specific binding, measured using 3 μM XAC (Tocris).

### Immunocytochemistry

The immunocytochemical detection of A_2A_R in individual glutamatergic and GABAergic nerve terminals was conducted as previously described (Canas et al., 2014; Kaster et al., 2015) by double-labeling with goat anti-A_2A_R (1:200, Santa Cruz Biotechnology), together with either guinea-pig anti-vGluT1 (1:500, Synaptic Systems) or guinea-pig anti-vGAT (1:200, Synaptic Systems) antibodies, followed by incubation with Alexa Fluor-labeled secondary antibodies (1:500, Invitrogen). The preparations were examined under a Zeiss Imager Z2 fluorescence microscope and each coverslip was analyzed by counting three different fields and in each field an average of 500 individualized elements (Canas et al., 2014).

### Western blot analysis

Western blot analysis was conducted by SDS-PAGE using synaptosomal membranes to evaluate synaptic markers, using antibodies against syntaxin-I (1:5000; Sigma), SNAP25 (1:2000; Sigma) and vGluT1 (1:5000; Millipore Bioscience Research Reagents), as previously described (Canas et al., 2014; Kaster et al., 2015). Membranes were reprobed for α-tubulin (1:1000; Abcam) as a loading control.

### Glutamate release

Rat hippocampal slices (Costenla et al., 2011) obtained at 3 h after administering saline or kainate (10 mg/kg) to trigger Racine-stage 4–5 convulsions, recovered for 45 min, before being loaded for 15 min at 37°C with ^3^H-glutamate (2 µM; specific activity, 0.319 Ci/mol) in the presence of aminooxyacetic acid (100 µM; Sigma). Slices were then superfused (0.7 ml/min) for 10 min and next stimulated for 3 min with 20 mM K^+^. The tritium content of the collected effluent samples and of the harvested slices was counted and the fractional release (FR%) calculated for each sample as the percentage of ^3^H-glutamate content in the slice (Lopes et al., 2002).

### Neuronal culture in microfluidic chambers

Hippocampal neurons were cultured from 17- to 19-d-old Wistar rat embryos and plated on microfluid chambers as previously described (Taylor et al., 2005; Pinto et al., 2016). The dissociated neurons were placed in one chamber and only the axons can grow (around DIV4/5) through the microgrooves into the opposite chamber. Neurons were cultured at 37°C in a 5% CO_2_ humidified atmosphere in Neurobasal medium with B_27_ supplement, glutamate (25 mM), glutamine (0.5 mM), and gentamicin (0.12 mg/ml).

### PCR analysis

Total RNA was extracted from the hippocampus with MagNA Lyser (Roche) to calculate the mRNA expression of A_2A_R, CD11b and Iba1 by real-time PCR using a SYBR Green I kit (Roche) and the comparative cycle threshold method with glyceraldehide-3’-phosphate dehydrogenase as housekeeping, as previously described (Costenla et al., 2011; Rebola et al., 2011). Non-quantitative PCR to detect A_2A_R mRNA was also conducted in cDNA samples from synaptosomes (previously incubated with RNase for 30 min at 37°C) or striatal tissue from rats, or a scraped collection of axon terminals or of cell bodies from rat hippocampal neurons cultured in different microfluidic chambers, using histone-1 mRNA as nuclear control and β-actin mRNA as synaptic control.

### Electrophysiological recordings

Recordings of excitatory synaptic transmission and plasticity were performed in superfused hippocampal slices (400 μm thick), as previously described (Costenla et al., 2011; Kaster et al., 2015). Briefly, Schaffer fibers were stimulated every 15 s to evoked field EPSPs (fEPSPs) recorded in the CA1 stratum radiatum to measure the fEPSP slope. Long-term potentiation (LTP) was induced with a high-frequency train (100 Hz for 1 s) and was quantified as the percentage change between the fEPSP slopes 60 min after and 15 min before the train. LTP amplitude was compared in different slices from the same animal in the absence and presence of a supramaximal concentration (50 nM) of SCH58261.

### Experimental design and statistical analyses

Data are mean ± SEM values. Data with one condition and one variable (effects of A_2A_R antagonist or of kainate treatment) were analyzed with Student’s *t* test. Data with more than one variable (effect of A_2A_R blockade on kainate treatments) were analyzed with a two-way ANOVA followed by Newman–Keuls *post hoc* tests with a significance level was 95%.

## Results

### A_2A_R blockade attenuates epileptogenesis on amygdala kindling

**Figure 1.**
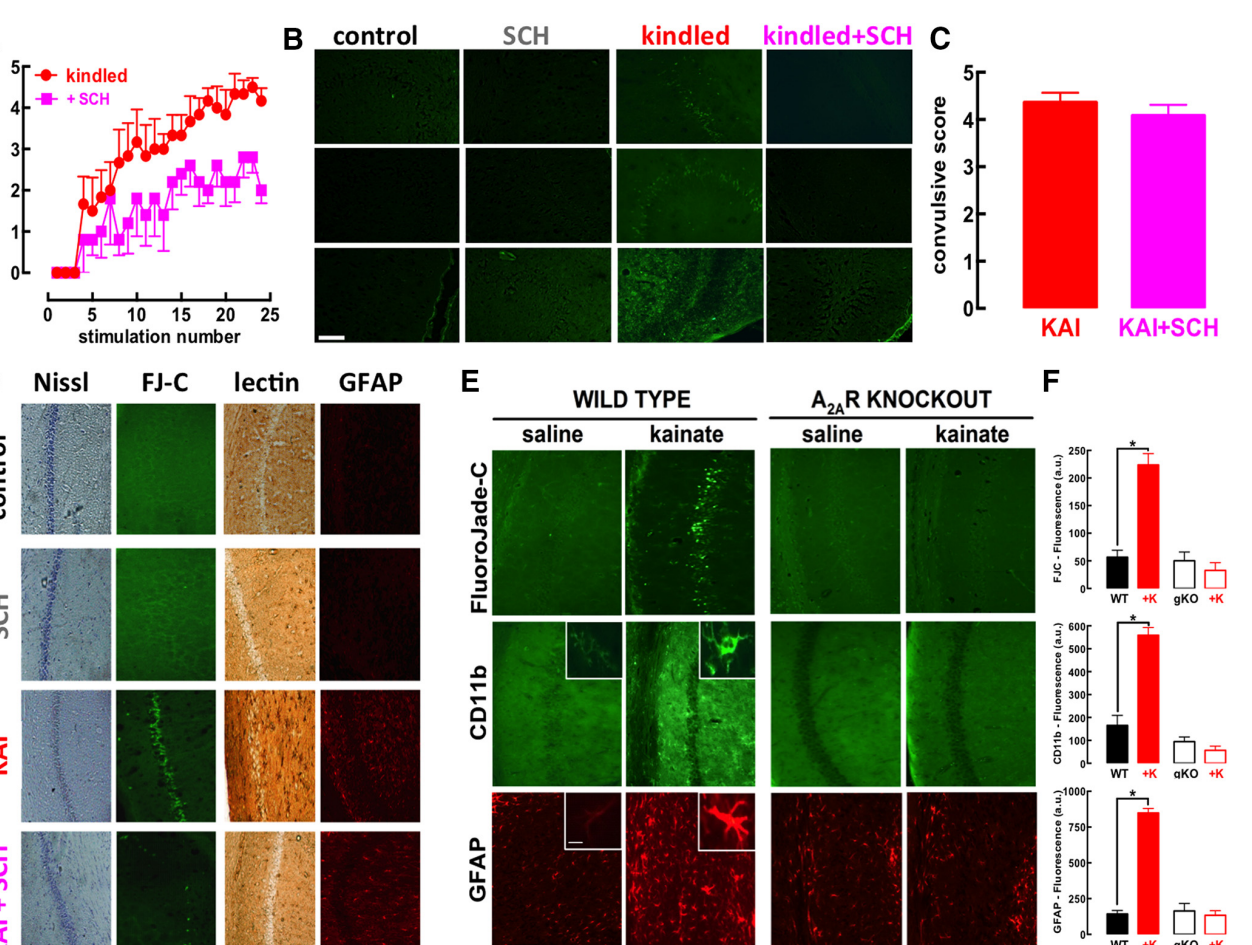
The pharmacological blockade or genetic elimination of adenosine A_2A_R prevents hippocampal damage caused by either amygdala kindling or kainate administration, while only attenuating the evolution of convulsions. ***A***, Male Wistar rats were implanted with electrodes in the amygdala and stimulation twice daily progressively increased the severity of convulsions until triggering a fully kindled state (*n* = 7), which was reduced by a selective A_2A_R antagonist SCH58261 (0.05 mg twice daily; *n* = 8). ***B***, Rats were killed 5 d after reaching fully kindled state and only the kindled rats treated with saline displayed degenerated cells, identified by FluoroJade-C labeling, in all hippocampal fields, whereas neither control nor SCH58261-treated rats, irrespective of being kindled or not, displayed FluoroJade-C staining. ***C***, On intraperitoneal administration of kainate (KAI, 10 mg/kg; *n* = 11), rats treated with 0.05 mg/kg of SCH58261 (KAI+SCH, *n* = 12) displayed a similar pattern of acute convulsions (within 15 min and lasting no >75 min after KAI). ***D***, However, SCH58261 prevented the histologic modifications observed in the hippocampus 24 h after kainate administration, namely, the dispersion of pyramidal cell layer with Nissl staining, the appearance of ruptured cells stained with FluoroJade-C (FJ-C), the modification of microglia staining with tomato lectin, and the increase of the number and density of GFAP-stained element compatible with astrogliosis. ***E***, ***F***, The administration of kainate (35 mg/kg, sc) to wild-type (WT) C57Bl6 mice (*n* = 11) triggered a convulsive period followed by the appearance of degenerated cells stained with FluoroJade-C together with a microgliosis and astrogliosis concluded from the altered staining of hippocampal sections with CD11b and GFAP, respectively; notably, the same exposure to kainate (+K) of littermates with a genetic deletion of A_2A_R (A_2A_R-KO, gKO) triggered a similar intensity of convulsions, which did not evolve into an evident pattern of neurodegeneration, microgliosis, or astrogliosis in the hippocampus after 24 h (*n* = 10). Calibration bars in each photograph are 100 µm, except the insets, which display higher magnifications of either astrocytes or microglia (calibration bar = 10 µm). Data are mean ± SEM; *p < 0.05 between bars or versus control (saline).

Rats subjected to daily electrical stimulation of the amygdala slowly began displaying convulsions that escalated over continuous sessions of electrical stimulation (kindling; Fig. 1*A*). Both the onset and evolution of convulsions was attenuated in rats treated with the selective A_2A_R antagonist SCH58261 (0.05 mg/kg; Fig. 1*A*). After 24 d of stimulation, the hippocampus displayed neurodegenerative features, as heralded by the presence of cells stained with FluoroJade-C in all subregions only in kindled but not in sham-operated (control) or SCH58261-treated rats, irrespective of being kindled or not (Fig. 1*B*). Thus, A_2A_R blockade blunted convulsions-induced neurodegeneration while it only attenuated the escalating convulsive profile; this suggests that this prominent neuroprotection might underlie the anti-epileptogenic effect of A_2A_R antagonists previously reported in other animal models of epilepsy (El Yacoubi et al., 2009; Li et al., 2012). However, since tinkering with A_2A_R affected both abnormal excitability and neuronal damage, which are tightly intertwined, we had to turn to another model of epilepsy allowing a separation of abnormal excitability from the subsequent damage.

### A_2A_R blockade does not affect kainate-induced convulsions but prevents subsequent neurodegeneration

To confirm this hypothesis, we used kainate administration, which triggers a period of intense convulsions followed by neuronal damage evident after 24 h and evolving with time (Sperk et al., 1983). In contrast to the amygdala kindling model, the kainate model of convulsions, allows to temporally disentangle convulsions and neurodegeneration. Kainate (10 mg/kg, i.p.) triggered convulsions reaching a maximal intensity of 4–5 in the Racine’s scale within 40–60 min, which had similar intensities in saline- and SCH58261-treated rats (Fig. 1*C*). This similar duration and intensity of convulsions tentatively allows isolating the effect of A_2A_R on neurodegeneration. Kainate damaged hippocampal pyramidal neurons, visible after 24 h as a decreased Nissl staining and appearance of FluoroJade-C-stained cells in CA1 area (Fig. 1*D*), which was more evident than in CA3 region (data not shown; see also Sperk et al., 1983), together with an astrogliosis (4-fold more GFAP-stained profiles, *n* = 9) and microgliosis (increased number of profiles labeled with tomato lectin displaying thicker processes, i.e., “activated” microglia; Dalmau et al., 2003). These modifications were more pronounced 7 d after kainate injection (data not shown), in accordance with the evolution of limbic lesions following convulsions (Sperk et al., 1983). SCH58261 (0.05 mg/kg) virtually eliminated kainate-induced neuronal damage and glia-related modifications, indicative of astrogliosis and microgliosis in the hippocampus (Fig. 1*D*).

We next ascertained whether A_2A_R also control convulsions-induced neurodegeneration in mice. Differently from rats, mice required a subcutaneously treatment with a higher dose of kainate (35 mg/kg) to convulse with a maximal intensity of 4–6 in the modified Racine’s scale within 45–70 min. Again, in all experimental groups, we always confirmed that mice displayed similar intensity and duration of convulsions to tentatively isolate the effect of A_2A_R on neurodegeneration. Kainate damaged hippocampal pyramidal neurons, visible after 24 h as a decreased Nissl staining, as well as an appearance of FluoroJade-C-stained cells in the Cornu Ammonis. Notably, mice with genetic A_2A_R deletion (global A_2A_R-KOs) displayed a convulsive profile (4.30 ± 0.30, *n* = 10) similar (*F*_(1,37)_ = 2.62; *p* = 0.11) to wild-type littermates (4.90 ± 0.23, *n* = 10), but did not display the kainate-induced neuronal damage, astrogliosis, or microgliosis that were present in wild-type mice (Fig. 1*E*,*F*). Similarly, SCH58261-treated mice did not display kainate-induced damage although they convulsed similarly to vehicle-treated mice (data not shown).

### A_2A_R are upregulated in glutamatergic synapses through a local translation

**Figure 2.**
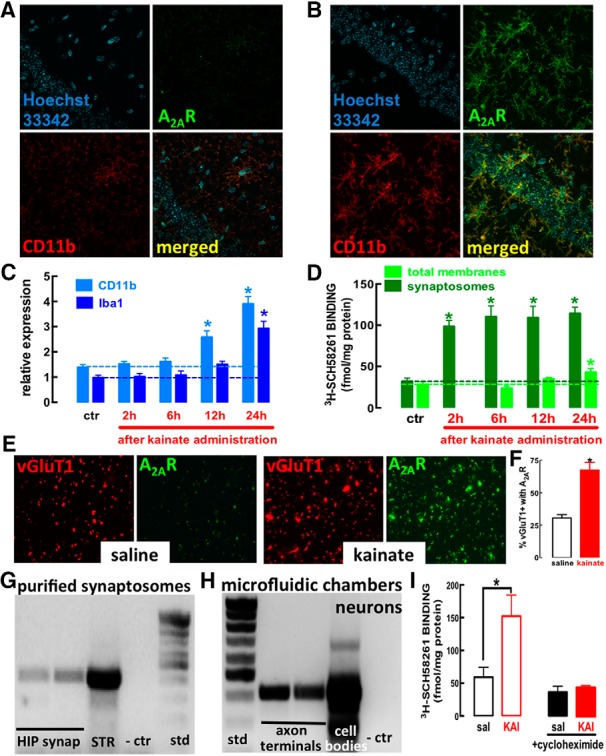
A convulsive period triggers an early upregulation of A_2A_R in glutamate synapses, likely involving a local translation of synaptic A_2A_R mRNA, and a delayed upregulation of A_2A_R in modified microglia cells in the hippocampus. ***A***, The immuno-density of the microglia marker CD11b and of A_2A_R were low in hippocampal sections from saline-treated mice, whereas they were increased and colocated circa 22 h after a convulsive period triggered by the subcutaneous injection of 35 mg/kg kainate (***B***; confocal images representative of *n* = 3 mice per group). ***C***, Kainate-induced convulsions triggered an increase of mRNA levels of CD11b and Iba1, characteristic of reactive microglia, only after 12–24 h in mouse hippocampal extracts (*n* = 6 for each time point). ***D***, In contrast, an increased A_2A_R density was observed in synaptosomes (purified synapses) as soon as 2 h after kainate injection, whereas an A_2A_R binding density was only upregulated in total membranes after 24 h in the hippocampus (*n* = 5 for each determination, except *n* = 6 at 24 h). ***E***, ***F***, Kainate-induced A_2A_R upregulation was evident in glutamate synapses, as gauged by the kainate-induced increased A_2A_R immunoreactivity in hippocampal synaptosomes immuno-positive for vesicular glutamate transporter type 1 (vGluT1; *n* = 6). ***G***, A_2A_R mRNA was identified in purified synaptosomes from the hippocampus (HIP), similarly to its presence in rat striatal extracts (STR) of the rat brain (*n* = 3). ***H***, The synaptic localization of A_2A_R mRNA was confirmed in a pure axonal preparation from hippocampal neurons cultured in microfluidic chambers, which ensures a physical segregation of axon terminals and cell bodies (*n* = 2). ***I***, The contribution of the local translation of synaptic A_2A_R mRNA for the kainate-induced upregulation of A_2A_R was concluded by the ability of cycloheximide (*n* = 4) to prevent the increase of A_2A_R binding density in synaptosomes exposed for 2 h to 5 μM kainate (*n* = 5). Data are mean ± SEM; *p < 0.05 between bars or versus control (saline, sal).

Neuroinflammation and glutamate excitotoxicity are involved in epilepsy-associated neurodegeneration (Coyle, 1987; Devinsky et al., 2013) and brain insults can upregulate A_2A_R in both microglia and synapses (Cunha, 2016), whereas the upregulation of A_2A_R in astrocytes occurs later in processes of neurodegeneration (Orr et al., 2015; Ogawa et al., 2018). Thus, we tested whether convulsions upregulated A_2A_R in microglia and/or in synapses. At 24 h after kainate administration, mouse hippocampal cellular elements labeled with the microglia marker CD11b displayed increased immunoreactivity for both CD11b and A_2A_R (Fig. 2*A*,*B*), whereas there was no observable A_2A_R immunoreactivity in hippocampal sections collected from A_2A_R-KO mice challenged with kainate (data not shown). However, PCR analysis of hippocampal extracts from kainate-treated mice revealed a different time course for the upregulation of A_2A_R and for the microglia markers CD11b and Iba1 (Fig. 2*C*,*D*), which are rapidly upregulated by triggers of neuroinflammation such as LPS, to inform on dynamic adaptive changes of microglia function (Rebola et al., 2011). CD11b and Iba1 mRNA levels only increased after 12–24 h (Fig. 2*C*), whereas A_2A_R binding density increased in hippocampal synaptic membranes as early as 2 h (Fig. 2*D*). This suggests that A_2A_R upregulation might occur in cellular compartments other than microglia. In fact, 2 h after kainate administration, an early A_2A_R upregulation was evident in glutamatergic synapses, as gauged by increased A_2A_R immunoreactivity in vGluT1-positive synaptosomes of kainate-treated versus saline-injected mice (*t* = 5.788; df = 10; *p* = 0.0002; Fig. 2*E*,*F*). This A_2A_R upregulation did not occur in GABAergic synapses identified as vGAT-positive (*t* = 0.232; df = 10; *p* = 0.82).

The speed of this synaptic A_2A_R upregulation and the distance between synapses and the nucleus in pyramidal hippocampal neurons prompted a possible involvement of local translation within synapses. This would imply the presence of A_2A_R mRNA in synapses. This was identified by qPCR in hippocampal synaptosomes (Fig. 2*G*) and confirmed in axonal ends of hippocampal neurons cultured in microfluidic chambers (Fig. 2*H*), which physically separate cell bodies from axon terminals (Taylor et al., 2005; Pinto et al., 2016). Moreover, exposure of synaptosomes (purified synapses, without nuclei) to kainate (5 μM, for 2 h) increased A_2A_R binding density (*t* = 2.609; df = 8; *p* = 0.031), an effect prevented by 100 μM cycloheximide, a protein synthesis inhibitor (*t* = 0.7845; df = 6; *p* = 0.463; Fig. 2*I*). This local synaptic translation of A_2A_R mRNA provides a rationale for the rapid A_2A_R upregulation selectively in synapses shortly after noxious stimuli.

### Convulsions bolster A_2A_R-mediated potentiation of glutamatergic synapse function

**Figure 3.**
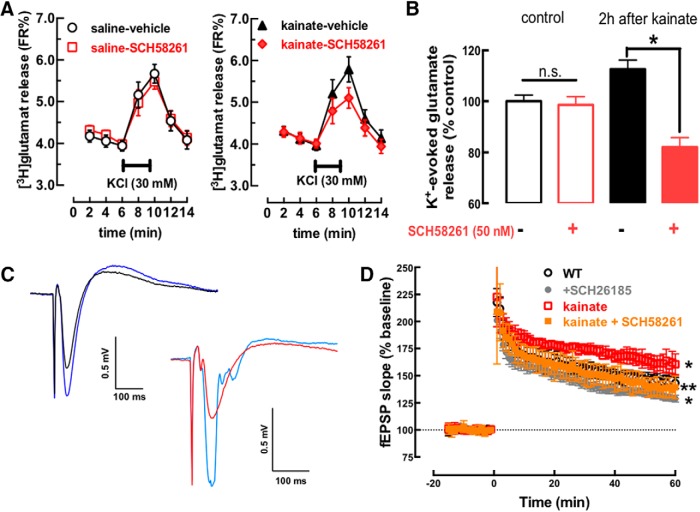
A convulsive period bolsters the function of synaptic A_2A_R in glutamatergic synapses. ***A***, ***B***, A_2A_R blockade with SCH58261 (50 nM) did not modified the depolarization-evoked release of glutamate (i.e., on augmentation of extracellular K^+^ as indicated by the horizontal insert line) from hippocampal synaptosomes from saline-treated mice (left panel, *n* = 8), but increased glutamate release from synaptosomes collected from convulsing rats 2 h after kainate administration (10 mg/kg, i.p.; right panel, *n* = 6). ***C***, ***D***, The amplitude of LTP, triggered by high-frequency stimulation (100 Hz for 1 s) of afferent Schaffer fibers, was larger in CA1 synapses from hippocampal slices collected 4 h after the administration of kainate (*n* = 6) than in saline-treated mice (*n* = 5). ***C***, Pairs of superimposed fEPSP recorded 10 min before (black and red traces) and 60 min after the high-frequency train (dark or light blue traces) in slices from saline-treated mice (left pair) or 4 h after kainate administration (right pair). ***D***, Time course of average fEPSPs before and after application of the high-frequency train (at time 0) in slices from control mice (black symbols), the aberrantly large LTP in slices collected 4 h after kainate administration (red symbols) and the ability of 50 nM SCH58261 to bring LTP amplitude back to control levels in these slices collected 4 h after kainate injection (orange symbols). This indicates that convulsions-induced aberrant synaptic plasticity is due to overfunctioning of A_2A_R. Data are mean ± SEM; *p < 0.05 versus control (saline in ***B***; WT – wild type in ***D***); **p < 0.05 comparing kainate versus kainate+SCH58261 in ***D***.

To probe the impact of A_2A_R upregulation, we first compared glutamate release from hippocampal slices collected from rats killed 3 h after the administration of either vehicle or kainate (Fig. 3*A*). SCH58261 (50 nM) inhibited the K^+^ (20 mM)-induced glutamate release in the kainate-treated group (*t* = 2.735; df = 7; *p* = 0.029), but not in the saline group (*t* = 1.435; df = 7; *p* = 0.195; Fig. 3*A*,*B*), indicating a greater tonic activation of A_2A_R bolstering glutamate release after kainate-induced convulsions. We next evaluated the A_2A_R-mediated selective control of LTP in CA1 pyramid synapses (Costenla et al., 2011). LTP amplitude was larger in slices 4 h after kainate treatment than in saline-treated mice (*F*_(1,20)_ = 11.65; *p* = 0.0028; Fig. 3*C*,*D*). Moreover, A_2A_R blockade caused a larger decrease of LTP amplitude (37.6 ± 4.4%) in slices from kainate- than saline-treated mice (25.6 ± 3.9%; interaction: *F*_(1,20)_ = 5.01; *p* = 0.048; Fig. 3*D*), bringing LTP amplitude in slices from kainate-treated mice (38.30 ± 3.35% above baseline) to values close to control (43.78 ± 2.08%). This indicates that convulsions trigger a rapid glutamatergic hyperfunction through an increased A_2A_R modulation.

**Figure 4.**
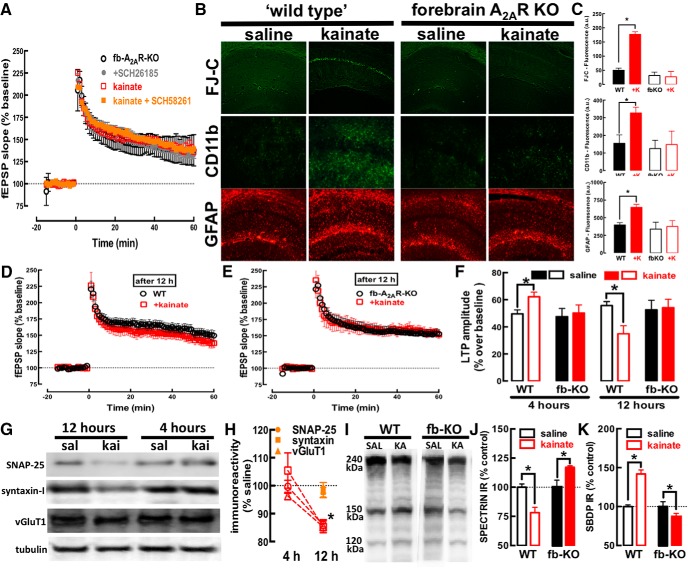
Neuronal A_2A_R are critical to convert the initial convulsions-induced glutamate hyperfunctioning into a subsequent dysfunction and loss of glutamate synapses with the activation of calpains. ***A***, ***B***, Absence of alteration of the high-frequency train (100 Hz for 1 s, applied at time 0)-induced LTP of fEPSP recorded in the CA1 stratum radiatum on stimulation of afferent Schaffer fibers in hippocampal slices collected 4 h after the induction of a convulsive period on subcutaneous administration of 35 mg/kg kainate to mice with a genetic deletion of A_2A_R selectively in forebrain neurons (fb-A_2A_R-KO, *n* = 6). ***B***, ***C***, Likewise, fb-A_2A_R-KO mice analyzed 24 h after the injection of kainate did not display neuronal damage (FluoroJade-C, FJ-C), microgliosis (CD11b immunoreactivity) or astrogliosis (GFAP immunoreactivity), which was present in “wild-type” littermates (*n* = 8–10 mice per group). The conversion from the initial convulsions-induced hyperfunctioning of glutamate synapses into neurodegeneration first involved a synaptic dysfunction, as shown (***D***) by the lower amplitude of LTP in hippocampal slices collected 12 h after the administration of kainate to trigger a convulsive period within the first 75 min (red symbols) compared to saline-treated mice (black symbols; *n* = 10). ***E***, The kainate-induced reduction of LTP amplitude at 12 h is not present in fb-A_2A_R-KO mice (*n* = 7). ***F***, Summary of the time-dependent evolution of the impact of a kainate-induced convulsive period on hippocampal synaptic plasticity: LTP was first bolstered (at 4 h) and later depressed (at 12 h) in a manner strictly dependent on neuronal A_2A_R (lack of alterations in fb-A_2A_R-KO). ***G***, ***H***, The evolution from overexcitation to decreased synaptic plasticity caused by kainate-induced convulsions was associated with a loss of synaptic markers, namely, SNAP-25, syntaxin-I and vesicular glutamate transporters type 1 (vGluT1), which was seen 12 h after kainate administration (*n* = 4), but not after 4 h in wild-type mice (WT; open symbols, *n* = 4) or in fb-A_2A_R-KO mice (filled symbols, *n* = 4). ***I–K***, This putative synaptotoxicity likely involved the recruitment of calpains, which was strictly dependent on the presence of neuronal A_2A_R: in fact, kainate triggered a decreased immunoreactivity of the calpain substrate, spectrin (***I***, ***J***), and a parallel increase of the immunodensity of the calpain-derived spectrin breakdown degradation products (SBDP-145–150 kDa) in wild-type but not in fb-A_2A_R-KO mice (***I***, ***K***; *n* = 5 in each group). Data are mean ± SEM; *p < 0.05 between bars or between kainate and saline (SAL) or versus control (100%).

To confirm the selective involvement of neuronal A_2A_R, we exploited forebrain A_2A_R-KO mice, which lack A_2A_R selectively in principal neurons of the forebrain (Shen et al., 2008). In forebrain A_2A_R-KO mice, kainate failed to alter LTP amplitude (*F*_(1,17)_ = 0.028; *p* = 0.86) and SCH58261 (50 nM) was also devoid of effects on LTP amplitude (*F*_(1,17)_ = 0.457; *p* = 0.50; Fig. 4*A*). Additionally, hippocampal cellular damage, astrogliosis, and microgliosis were present in wild-type littermates but were not observed in forebrain A_2A_R-KO mice 24 h after kainate administration (Fig. 4*B*,*C*), although the intensity of convulsions in wild-type mice (4.80 ± 0.20, Racine’s modified scale) was similar (*F*_(1,38)_ = 0.1293; *p* = 0.72) to that of forebrain A_2A_R-KO mice (4.70 ± 0.20). These findings directly support a key role of neuronal A_2A_R in the control of neurodegeneration triggered by a convulsive episode.

### A_2A_R-induced glutamate hyperfunction triggers a subsequent dysfunction of glutamate synapses

To clarify the evolution from an initial A_2A_R-mediated bolstering of glutamatergic activity (at 4 h) into subsequent neuronal damage (at 24 h), we characterized alterations present 12 h after kainate administration. The kainate-induced increase of LTP amplitude at 4 h (Fig. 3*D*) was transformed into an inhibitory effect at 12 h (*t* = 2.887; df = 14; *p* = 0.012; Fig. 4*D*,*F*), which was also abolished (*t* = 0.1666; df = 10; *p* = 0.87) in forebrain A_2A_R-KO mice (interaction kainate × genotype: *F*_(1,24)_ = 4.588; *p* = 0.040; Fig. 4*E*,*F*). In parallel, at 4 h, there was no significant alteration in the density of different synaptic markers (SNAP25: *t* = 0.580; df = 6; *p* = 0.58; syntaxin: *t* = 0.246; df = 6; *p* = 0.81; vGluT1: *t* = 1.423; df = 6; *p* = 0.20; Fig. 4*G*,*H*), contrasting to the decreased density at 12 h of SNAP25 (*t* = 7.313; df = 6; *p* = 0.0003), syntaxin (*t* = 11.37; df = 6; *p* < 0.0001), and vGluT1 (*t* = 6.912; df = 6; *p* = 0.0005) in the hippocampus of kainate- versus saline-treated mice (Fig. 4*G*,*H*), which was absent in forebrain A_2A_R-KO mice (SNAP25: *t* = 0.298; df = 6; *p* = 0.78; syntaxin: *t* = 1.227; df = 6; *p* = 0.27; vGluT1: *t* = 1.524; df = 6; *p* = 0.18; Fig. 4*H*). However, although the neurochemical data indicated the presence of synaptotoxic alterations, there was no evidence of overt major morphologic changes in hippocampal section at 12 h after kainate administration, which displayed a lack of FluoroJadeC staining and no evident alteration of the pattern of GFAP or CD11b immunoreactivity.

### A_2A_R control the activity of calpains

Calpains are implicated in epilepsy-associated neurodegeneration (Araújo et al., 2008). Indeed convulsions increased calpain activity in the hippocampus (Fig. 4*I*), as gauged by the decreased density of the calpain substrate spectrin (Fig. 4*I*,*J*; *F*_(1,16)_ = 20.89; *p* = 0.0003) paralleled by the increased density of calpain-derived spectrin breakdown degradation products (SBDP-145–150 kDa; *F*_(1,16)_ = 8.838; *p* = 0.009), rather than caspase-3-derived SBDP-120 kDa (Fig. 4*I*,*K*). Importantly, both the decrease of spectrin (interaction kainate × genotype: *F*_(1,16)_ = 10.98; *p* = 0.006) and the increase of SBDP-145–150 kDa densities (interaction kainate × genotype: *F*_(1,16)_ = 36.41; *p* < 0.0001) were not observed in forebrain A_2A_R-KO mice (Fig. 4*I*,*K*).

### Therapeutic prospects of A_2A_R antagonists to control convulsions-induced neurodegeneration

**Figure 5.**
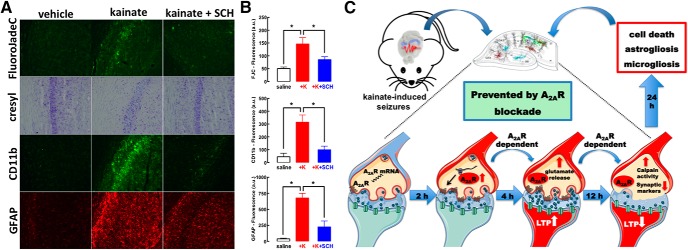
The time window between kainate-induced convulsions and synaptotoxicity and neurodegeneration offers a therapeutic window for A_2A_R antagonists to prevent convulsions-induced neurodegeneration. ***A***, ***B***, The selective A_2A_R antagonist SCH58261 (SCH, 0.05 mg/kg) applied intraperitoneally 4 h after kainate-induced convulsions was therapeutically effective to abrogate the emergence of neuronal damage (Nissl staining and FluoroJade-C), microgliosis (CD11b immunoreactivity), or astrogliosis (GFAP immunoreactivity) 24 h after kainate (K) administration (*n* = 5 mice per group). The data are mean ± SEM; *p < 0.05 between the indicated bars. ***C***, The pharmacological or genetic blockade of A_2A_R did not affect kainate-induced convulsions but dampened the subsequent neurotoxicity. This neurotoxicity began with a rapid A_2A_R upregulation in glutamatergic synapses (within 2 h), through local translation of synaptic A_2A_R mRNA. This bolstered A_2A_R facilitation of glutamate release and of LTP in CA1 synapses (4 h), triggered a subsequent synaptotoxicity, heralded by decreased synaptic plasticity and loss of synaptic markers coupled to calpain activation (12 h), that predated overt neuronal loss accompanied by astrogliosis and microgliosis (24 h). All modifications were prevented by selective A_2A_R deletion in forebrain neurons. Overall, this shows that synaptic A_2A_R critically control the development of convulsions-induced neurodegeneration.

The time course of A_2A_R-mediated control of glutamate synapses and their subsequent degeneration suggests that there might be a time window for intervention after convulsions to mitigate neurodegeneration by blocking A_2A_R. Indeed, SCH58261 (0.1 mg/kg) administered 4 h after kainate-induced convulsions (all mice reaching stages 4–5) decreased neuronal damage and microgliosis and attenuated astrogliosis (Fig. 5*A*,*B*). This clearly indicates that A_2A_R have a particular role on the evolving processes of neurodegeneration after the convulsive period, which was anticipated based on the lack of alteration of convulsive activity after kainate administration, although the behavioral output of convulsions might not ensure a lack of alteration of any neurophysiological mechanism involved in the insult.

## Discussion

The present study shows that A_2A_R are paramount to link convulsions to neurodegeneration. We temporally disentangled different events triggered by kainate-induced convulsions in hippocampal tissue after the extinction of convulsions (Fig. 5*C*): the first observed event was the upregulation of A_2A_R in synapses, namely, in glutamatergic synapses (within 2 h); this was accompanied by an early increase of glutamatergic activity (within 4 h), typified by increased glutamate release and larger synaptic plasticity; in accordance with glutamate-mediated excitotoxicity being a trigger of neurodegeneration (Lipton and Rosenberg, 1994), we observed a later decrease of synaptic plasticity and loss of synaptic markers but no overt neurotoxicity (within 12 h) coupled to a calpain activation and overt neuronal damage (within 24 h). Our therapeutic-like intervention after the termination of convulsions provides a proof-of-concept to renforce our contention that A_2A_R are selectively involved in the control of neurodegeneration after seizures. However, we have not detailed the window of opportunity for intervention with A_2A_R antagonists, to define whether A_2A_R only control the initial process of excitotoxicity (seen at 6 h, without evidence of synaptotoxicity or overt neurotoxicity) and/or the process of synaptotoxicity (seen at 12 h without evidence of overt neurotoxicity) and the process of overt neurodegeneration (seen at 24 h).

We evaluated kainate-induced toxicity using three different readouts previously described to reflect kainate-induced hippocampal toxicity, namely, cell damage, astrogliosis, and microgliosis (Sperk et al., 1983; Pitkänen and Sutula, 2002; Benkovic et al., 2004). All these degenerative features were prevented by pharmacological or genetic A_2A_R inactivation in both rats and mice. This provides strong evidence that the tonic A_2A_R activation by endogenous adenosine is crucial to express hippocampal damage following kainate-induced convulsions. In contrast, the role of A_2A_R on behavioral seizures is, at best, disputable (El Yacoubi et al., 2001; Etherington and Frenguelli, 2004; Lee et al., 2004; Zeraati et al., 2006; Rosim et al., 2011; Li et al., 2012). Thus, similarly to the effects of caffeine (Rigoulot et al., 2003), A_2A_R selectively control hippocampal damage independently of their eventual ability to control the severity of convulsions. This is in accordance with the established role of A_2A_R in the control of NMDA receptors and synaptic plasticity processes, rather than to control excitability, which is a function instead fulfilled by A_1_R (for review, see Cunha, 2016), This prompts A_2A_R antagonists as novel “secondary neuroprotective agents” (Meldrum, 2002) arresting the limbic maladaptive plasticity underlying the progressive severity of epilepsy (Meldrum, 2002; Pitkänen and Sutula, 2002; Löscher and Brandt, 2010). However, future studies should evaluate if the manipulation of A_2A_R might also alleviate behavioral dysfunction often emerging after seizures, such as cognitive impairments that are controlled by A_2A_R in different brain disorders (for review, see Cunha, 2016).

Although A_2A_R are located in neurons as well as in glia (Cunha, 2016), the use of forebrain A_2A_R-KO mice provided direct evidence that it is A_2A_R in neurons that critically control kainate-induced neurodegeneration. Thus, the deletion of neuronal A_2A_R is sufficient to fully account for the role of A_2A_R in the development of neurodegeneration following seizures and further studies should clarify if A_2A_R in microglia and/or astrocytes might also play an ancillary role in seizures-induced neurodegeneration. Cerebral cortical A_2A_R are most abundantly located in synapses (Rebola et al., 2005b) with a low density in physiologic conditions (Lopes et al., 2004), as expected from a receptor selectively involved in bolstering synaptic plasticity (Rebola et al., 2008; Costenla et al., 2011). Probably as an attempt to increase adaptability after injury, A_2A_R are upregulated after brain insults (Cunha, 2016), namely, in epilepsy models (Rebola et al., 2005a) and patients (Barros-Barbosa et al., 2016). The mechanisms linking brain insults to A_2A_R upregulation are unknown, since the regulation of the promoter(s) of the A_2A_R gene and of its numerous transcripts encoding A_2A_R are still poorly understood (Lee et al., 2003; Yu et al., 2004). The present study reveals some surprising novel findings. First, we identified A_2A_R transcripts in synapses, where a local translation seems sufficient to account for synaptic A_2A_R upregulation. Second, we found that synaptic A_2A_R upregulation on brain dysfunction is a rapid event, occurring in less than 2 h. A_2A_R upregulation is well positioned to trigger a transient hyperfunction of glutamatergic synapses, which is involved in the pathophysiology of most neurodegenerative disorders (Lipton and Rosenberg, 1994), since hippocampal A_2A_R bolster glutamate release (Lopes et al., 2002), NMDA receptor function (Rebola et al., 2008), and calcium influx in synapses (Gonçalves et al., 1997). Furthermore, we now show that A_2A_R activity is strictly required to trigger calpain activity that was previously shown to mediate kainate-induced neurodegeneration (Araújo et al., 2008).

These early synaptic modifications match the recognition of synapses as initial triggers of neurodegeneration in other neurodegenerative conditions such as Alzheimer’s disease (Selkoe, 2002), with a predominant early alteration of glutamatergic synapses (Kirvell et al., 2006; Canas et al., 2014). Accordingly, animal models and epileptic patients with sclerosis display a loss of synaptic markers (Looney et al., 1999; Zhang et al., 2014), in particular of glutamatergic markers (Alonso-Nanclares and De Felipe, 2005; van der Hel et al., 2009). This is compatible with the engagement of glutamate-mediated excitotoxicity, involving NMDA receptor activation and excessive calcium influx to activate calpains to destroy glutamatergic synapses before the emergence of overt neuronal death (Cunha, 2016), through mechanisms where glia cells likely participate (Pitkänen and Sutula, 2002; Devinsky et al., 2013).

This conclusion that A_2A_R activation is paramount for the development of convulsions-induced neurodegeneration prompts reevaluating the concept of adenosine as an anti-epileptic agent (Dragunow, 1988; Dunwiddie, 1999). The action of adenosine through A_1_R lowers seizure onset acting as anti-convulsive (Dragunow, 1988; Dunwiddie, 1999), but A_1_R undergo a rapid desensitization after convulsions (Young and Dragunow, 1994; Ochiishi et al., 1999; Rebola et al., 2003). The present observation that the tonic A_2A_R activation by endogenous adenosine plays a pivotal role for the expression of damage in hippocampal tissue after a convulsive period, shows that the role of adenosine in the control of epilepsy may be more complex than previously proposed. Thus, adenosine plays a bi-phasic role in the control of epilepsy, lessening convulsive episodes but bolstering subsequent damage. This newly identified selective role of A_2A_R in the control of the neurodegeneration that develops as a consequence but after the convulsive period (Sperk et al., 1983; Pitkänen and Sutula, 2002) heralds the concepts of A_2A_R blockade as a new therapeutic strategy to arrest the evolution of epilepsy. Indeed, A_2A_R blockade attenuated amygdala- or pentylenetetrazol-induced kindled seizures (El Yacoubi et al., 2009), two models of slowly developing convulsions. Furthermore, we now showed that the administration of a selective A_2A_R antagonist after the convulsive period was still effective to arrest the subsequent hippocampal damage. This is in agreement with the reported ability of A_2A_R antagonists to prevent the long-term development of behavioral abnormalities in adult rats after convulsions early in life (Cognato et al., 2010). Also, genetic polymorphisms of A_2A_R (ADORA2A) are associated with childhood encephalopathy resulting from biphasic seizures (Shinohara et al., 2013). Together, this evidence heralds the new concept that A_2A_R are paramount for the development of neurodegeneration after convulsions.

## Synthesis

Reviewing Editor: Maiken Nedergaard, University of Rochester Medical Center Decisions are customarily a result of the Reviewing Editor and the peer reviewers coming together and discussing their recommendations until a consensus is reached. When revisions are invited, a fact-based synthesis statement explaining their decision and outlining what is needed to prepare a revision will be listed below. Note: If this manuscript was transferred from JNeurosci and a decision was made to accept the manuscript without peer review, a brief statement to this effect will instead be what is listed below.

I find that the authors' response to the critique raised in the J Neuroscience submission is powerful and detailed. The changes made in the manuscript in response to the reviewers are more than sufficient. As the reviewing editor with expertise in the adenosine field, I do not find that additional reviews are needed. The manuscript is ready for publication.
